# Neuropilin-2 Is a Newly Identified Target of PAX8 in Thyroid Cells

**DOI:** 10.1371/journal.pone.0128315

**Published:** 2015-06-01

**Authors:** Valeria Lucci, Tina Di Palma, Mariastella Zannini

**Affiliations:** IEOS-CNR, Institute of Experimental Endocrinology and Oncology ‘G. Salvatore’-National Research Council, Naples, Italy; IPATIMUP/Faculty of Medicine of the University of Porto, PORTUGAL

## Abstract

PAX8 is a transcription factor essential for thyroid gland development, as well as for the maintenance of the thyroid differentiated state in the adult. In particular, PAX8 has been comprehensively shown to regulate genes that are considered markers of thyroid differentiation. However, a better knowledge of genes transcriptionally regulated by PAX8 is desirable to clarify its role in endocrine syndromes and cancer susceptibility. In order to further investigate PAX8 downstream targets, we recently performed a genome-wide expression analysis following PAX8 knockdown in FRTL-5 thyroid cells and Neuropilin-2 was identified as a potential transcriptional target of PAX8. In this study, we determined the role of the transcription factor PAX8 in the regulation of Neuropilin-2 expression. Indeed, in thyroid cells PAX8 directly binds the Neuropilin-2 promoter leading to its transcriptional repression. Interestingly, we observed an inverse correlation between the expression of PAX8 and Neuropilin-2 in thyroid carcinoma tissues and cell lines compared to non-tumor counterparts, suggesting a critical role of PAX8 in regulating Neuropilin-2 expression in vivo. Notably, ectopic overexpression of PAX8 in FB-2 thyroid cancer cells promotes Neuropilin-2 downregulation producing a significant reduction in cell proliferation, migration ability, and invasion activity and reverting the cell phenotype from mesenchymal to a more epithelial one. These findings uncover the novel interplay between PAX8 and Neuropilin-2, which is likely to be important in the pathogenesis of thyroid diseases.

## Introduction

Neuropilins (NRP1 and NRP2) are multifunctional single-spanning trans-membrane glycoproteins that play a central role in neuronal and blood vessel development as receptors for members of the class-3 semaphorin family (SEMAs) of axonal guidance factors and also for members of the vascular endothelial growth factor (VEGF) family of angiogenesis stimulators [1–5]. Neuropilins are expressed by a wide variety of cells including endothelial cells, neurons, pancreatic islet cells, hepatocytes, melanocytes, and osteoblast and often by malignant tumor cells. In addition, expression occurs in some epithelial cells of several organs (e.g., skin, breast, prostate, GI tract, lung, kidney and bladder) [6–10]. NRP1 and NRP2 have 44% sequence homology and share many structural and biological properties [7, 11–17]. Neuropilins (NRPs) are usually expressed as homodimers, but NRP1/NRP2 heterodimers also occur [18]. The importance of NRPs in development has been demonstrated in knockout mice. *Nrp1* knockout in the mouse is lethal at E10-12.5; the embryos die with several defects in cardiac and vascular development, as well as disorganization of the pathway and projection of nerve fibers [19–21]. In contrast, *Nrp2* knockout mice are viable but have decreased numbers of lymphatics and capillaries, and defects of the central and peripheral nervous system [22]. The embryos of *Nrp1* and *Nrp2* double-knockout mice exhibit more severe anomalies and die earlier than *Nrp1* single-knockout mice [23]. In cancer, NRPs have been linked to a poor prognosis, which is consistent with their numerous interactions with ligands and receptors that promote tumor growth, migration and invasion. Overexpression of NRP1 in prostate carcinoma, colon carcinoma and glioma cancer models induces tumor angiogenesis and promotes tumor progression [24–26]. Similarly, NRP2 promotes tumor growth and metastasis in pancreatic adenocarcinoma and colorectal cancer models [27, 28]. In cancer patients, expression of NRP1, NRP2 or both NRPs is often upregulated and is correlated with tumor aggressiveness and advanced disease stage [29–34]. Importantly, NRPs appear to promote EMT and the maintenance of an immature or cancer stem cell phenotype [15, 35, 36]. In particular, NRP2 is also known as a coreceptor for vascular endothelial growth factor (VEGF)-D, which is a well-known lymphangiogenic factor that plays an important role in lymph node metastasis of various human cancers, including papillary thyroid carcinoma (PTC) [37, 38].

Recently, we investigated the genome-wide effect of PAX8 silencing comparing the transcriptome of silenced versus normal FRTL-5 differentiated thyroid cells and NRP2 was identified among the up-regulated genes [39]. PAX8 is a member of the paired box (Pax) family of genes encoding DNA binding proteins involved in the regulation of the development of a variety of tissues in different species. During embryogenesis PAX8 is expressed not only in the thyroid but also in other tissues such as the metanephros, the midhindbrain boundary region as well as in the Müllerian duct [40, 41]. PAX8 plays an essential role in the differentiation of thyroid cells [42] and according to the phenotype of *Pax8* knockout mice it is responsible for the formation of the follicles of polarized epithelial thyroid cells. In mice where the Pax8 gene has been deleted, the thyroid gland is completely devoid of thyroid hormone-producing follicular cells. As a consequence, PAX8 deficient mice are deaf, growth retarded, ataxic, and do not survive weaning [43]. In addition, the analysis of the male and female reproductive system demonstrated that the *Pax8* ^-/-^ mice fail to develop correctly and are infertile [44, 45]. Mutations in the PAX8 gene in humans have been associated with congenital hypothyroidism. Patients carrying the mutations are affected by thyroid dysgenesis, indicating an important role for this gene in thyroid pathologies [46–49]. In addition to hypothyroidism, PAX8 has a role in a subset of renal, bladder, ovarian, pancreatic endocrine and thyroid neoplasm [50–54].

In the present study, we demonstrate that the transcription factor PAX8 is able to bind *in vivo* the NRP2 gene promoter and to negatively regulate its expression in thyroid cells. We also show that the downregulation of NRP2 mediated by PAX8 reduces cell proliferation and suppresses cell migration and invasion of thyroid cancer cells.

## Materials and Methods

### Selection of cases and collection of thyroid tissue samples

For quantitative real-time PCR (qRT-PCR) analysis, thyroid tumors were provided by Prof. D. Salvatore (Medical School, University of Naples Federico II). Neoplastic human thyroid tissues and normal adjacent tissue or the contralateral normal thyroid lobe were collected and the study protocol was approved by the University Ethics Committee of the University of Naples Federico II (Naples, Italy). Written informed consent from the donors was obtained for the use of this samples in research. The use of human biological materials in Italy is regulated by 95/46/EC and Italian law N.675 dated 31.12.1996. Italian Legislative Decree N.211 of the 24.06.2003 enforces the European Directive 2001/20/CE on Good Clinical Practice Italian Personal Data Protection Code n. 196, 2003.

### RNA extraction, cDNA preparation and qRT-PCR

Total RNA was extracted using TRIzol reagent (Invitrogen) and treated with RNase-free DNase I (Applied Biosystem). For qRT-PCR the cDNAs were synthesized using the iScript cDNA Synthesis kit (BIORAD, Hercules, CA). To design the oligo primers we used the Primer Express software and the primers sequences are reported in S1 Table. Real time-PCR analysis was performed using the IQ SYBR Green PCRMasterMix (BIORAD) in an iCycler IQ Real-time detection system (BIORAD). Each reaction was performed in duplicate and a melting analysis was performed at the end of the PCR run. To calculate the relative expression levels we used the 2^-DDCT^ method. RNA samples of mouse thyroid carcinomas were kindly provided by A. Fusco (University of Naples Federico II).

### Cell culture and transfection

The rat thyroid FRTL-5 cell line was used for this study (ATCC, cat. CRL-1468). FRTL-5 cells were maintained in Coon’s modified F-12 medium (Euroclone, Milano, Italy) supplemented with 5% newborn bovine serum (HyClone, Logan, UT) and a six-hormone mixture (6H) as previously described [55].

WRO, FB-2, FRO, Cal62 and BCPAP cells were grown in DMEM (EuroClone) supplemented with 10% (v/v) fetal calf serum (HyClone).

For stable transfection of FLAG-PAX8 [56] or pCEFL backbone vector, FB-2 cells were cultured in 100 mm plates and transfected with 2 μg of DNA. 48 h after transfection, selection with the specific antibiotic (0.8 μg/ml G418, GIBCO) was started. The expression of 3XFLAG-PAX8 was assessed by Western blot. Transfections were carried out with the LIPOFECTAMINE reagent (Lifetechnologies) according to the manufacturer's directions.

### Protein extracts and immunoblotting

Cells were washed twice with ice-cold phosphate-buffered saline (PBS) and lysed in JS buffer containing 50 mM Hepes pH 7.5, 150 mM NaCl, 5 mM EGTA pH 7.8, 10% glycerol, 1% Triton, 1.5 mM MgCl2, 1 mM dithiothreitol (DTT), 1 mM phenylmethylsulfonyl fluoride (PMSF). The protein concentration was determined using the Bio-Rad protein assay (Bio-Rad Laboratories, Inc., Hercules, CA). For Western blot analysis, proteins were separated on SDS–PAGE, gels were blotted onto Immobilon P (Millipore, Bredford, MA, USA) for 2 h and the membranes were blocked in 5% nonfat dry milk in Tris–buffered saline for 2 h or overnight before the addition of the antibody for 1 h. The primary antibodies used were: anti-GAPDH (Santa Cruz, CA), anti-PAX8 (kindly provided by R. Di Lauro) anti-Tubulin (Santa Cruz, CA) and anti-NRP2 (R&D SYSTEMS). The filters were washed three times in Tris–buffered saline plus 0.05% Tween 20 before the addition of horseradish peroxidase-conjugated secondary antibodies for 45 min. Horseradish peroxidase was detected with ECL (Pierce).

### RNA interference

SMARTpool interfering RNAs targeting rat PAX8 mRNA were obtained from Dharmacon Technologies. For each experiment 8·10^4^ cells/well were plated in 24-well plates and transfected with 100 nM siRNA or scrambled control RNA (scRNA) using DharmaFECT Transfection Reagent according to the manufacturer’s instructions. Cells were harvested 24h, 48h and 72 h after transfection and total RNA was prepared using Trizol (Invitrogen).

### Wound-healing, invasion and cell proliferation assays

To evaluate cell growth, FB-2, FB2-FLAG pool, FB2-P8cl25 and FB2-P8cl31 cells were plated at 8 x 10^4^ cells per 60-mm plate. The medium was changed every 24 h, and every 24 h cells were collected and counted.

Confluent FB-2, FB2-FLAG pool, FB2-P8cl25 and FB2-P8cl31 cells plated on tissue culture dishes were wounded by manual scratching with 200-μl pipette tip, washed with PBS and incubated at 37°C in complete media. At the indicated time points, phase contrast images at specific wound sites were captured. Cell invasion assay was examined using a reconstituted extracellular matrix (Matrigel; BD Biosciences). Filters (8 μm pore size) on the bottoms of the upper compartment of the transwells (6,5 mm; Corning) were coated with 2 mg/ml of matrigel. 2x 10^5^ cells were suspended in 100 μ l of DMEM with 0.2% FBS. The cells were then plated onto the coated wells and incubated at 37°C for 16 h. Medium in the lower compartment was supplemented with 10% FBS as a chemoattractant. Non-invading cells were removed from the top of the wells with a moistened cotton swab. Cells that penetrated the membrane were fixed with 11% glutaraldehyde and stained with 0.1% crystal violet. The quantification of the wound-healing and invasion assays is shown in S1 Fig. Wound closure was calculated as the distance covered by cells in relation to the initial wound diameter, as determined at 0 h. Wound closure is expressed as the percentage of the initial wound diameter at 0 h. Data are expressed as the mean ± SD (P < 0.01).

For the invasion assay, columns in the graph represent the analysis of the cell count (P < 0.05).

### ChIP assay

ChIP assay was performed as previously described [39].

Precleared chromatin from FRTL-5 cells was incubated with 1 μg of affinity-purified rabbit polyclonal antibody anti-PAX8 (kindly provided by Prof. R. Di Lauro), unrelated antibody (anti-Tubulin, Santa Cruz) or no antibody and rotated at 4°C for 16h. The immunoprecipitated DNA was analyzed by PCR using the following NRP2 primers: 5’-GACAGCTGGGGATCATCCAAC-3’ and 5’-TAAATTGTCCCCTGGCCACCC-3’.

### Indirect immunofluorescence and confocal scanning laser microscopy

FB-2, FB2-FLAG pool, FB2-P8cl25 and FB2-P8cl31 cells were plated and cultured on 12 mm diameter glass coverslips, washed with PBS and fixed for 20 min at room temperature with 3.7% of paraformaldehyde. After permeabilization with 0.2% of Triton X-100 in PBS, cells were blocked in 0.5% BSA in PBS and incubated for 1 h at room temperature with primary antibodies (fibronectin clone IST4 from Sigma-Aldrich, vimentin from Santa Cruz Biotechnology), followed by Alexa Fluor-594 goat anti-mouse IgG (Life technologies) for 30 min at room temperature. Cell nuclei were identified by Hoechst staining. Images were collected with a Zeiss LSM 510 confocal laser scanning microscope (Zeiss, Oberkochen, Germany), equipped with a 543-nm HeNe laser and a Plan-Apochromat 63x/1.4 oil immersion objective. Emitted fluorescence was detected using a LP 560 long pass filter for Alexa Fluor 594. High magnification images were collected as 1024 ×1024 × 32 voxel images.

## Results

### PAX8 binds the Neuropilin-2 promoter region in vivo

**Fig 1:**
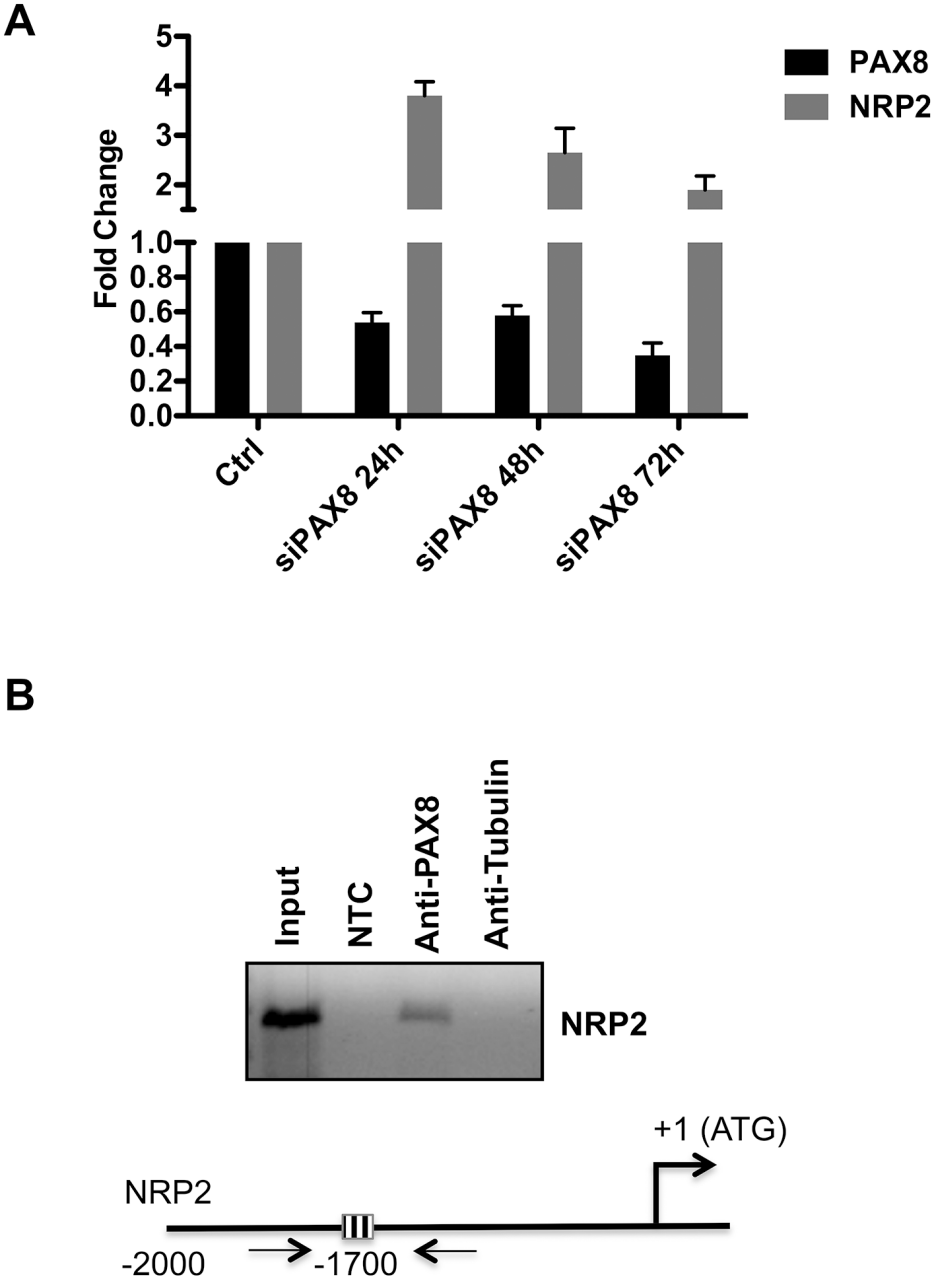
PAX8 directly modulates NRP2 gene expression. (A) FRTL-5 cells were transfected with a siRNA that specifically targets rat PAX8. PAX8 and Neuropilin-2 (NRP2) expression levels were measured on total RNA by qRT-PCR 24h, 48h and 72h after siRNA transfection. The values are means ± SD of three independent experiments in duplicate, normalized by the expression of β-actin and expressed as fold change with respect to the untransfected FRTL-5 cells, whose value was set at 1.0. Statistical analysis uses t test (p ≤ 0.05). (B) Chromatin extracted from cross-linked FRTL-5 cells was immunoprecipitated using in parallel an unrelated antibody (anti-tubulin) or an antibody against PAX8. The immunoprecipitates were analyzed by PCR with oligonucleotides corresponding to the rat NRP2 promoter region. Parallel PCR were performed with total input DNA obtained from unprecipitated aliquot of similarly treated chromatin sample and from no template control (NTC). Schematic representation of the upstream region of the rat NRP2 gene. PAX8-binding site is represented as a striped box.

To confirm the expression data obtained by the microarray analysis, we performed qRT-PCR to detect NRP2 transcript in RNA samples of FRTL-5 cells prepared 24h, 48h and 72h after PAX8 siRNA transfection. As shown in Fig 1A, the down-regulation of PAX8 in FRTL-5 cells well correlates with NRP2 increased expression levels. Subsequently, to investigate the ability of PAX8 to directly interact with the NRP2 promoter *in vivo*, a computational analysis using the MatInspector Software 8.0 was performed. We searched for PAX8 binding sites in a region of about 2000 bp of NRP2 5’-flanking region and the analysis showed the presence of PAX8 consensus sequences in the genomic region analyzed. To validate the prediction of the MatInspector analysis, we performed chromatin immunoprecipitation (ChIP) assays on genomic DNA of FRTL-5 cells using an antibody specific for PAX8. The enrichment of the endogenous NRP2 region was monitored by PCR amplification using specific primers (Fig 1B). Indeed, we show that the PAX8 antibody is able to immunoprecipitate the chromatin containing the NRP2 promoter. Taken together, the obtained results clearly highlight that NRP2 could be considered a novel and direct target of PAX8.

### Lack of PAX8 significantly affects Neuropilin-2 expression in thyroid cells

**Fig 2:**
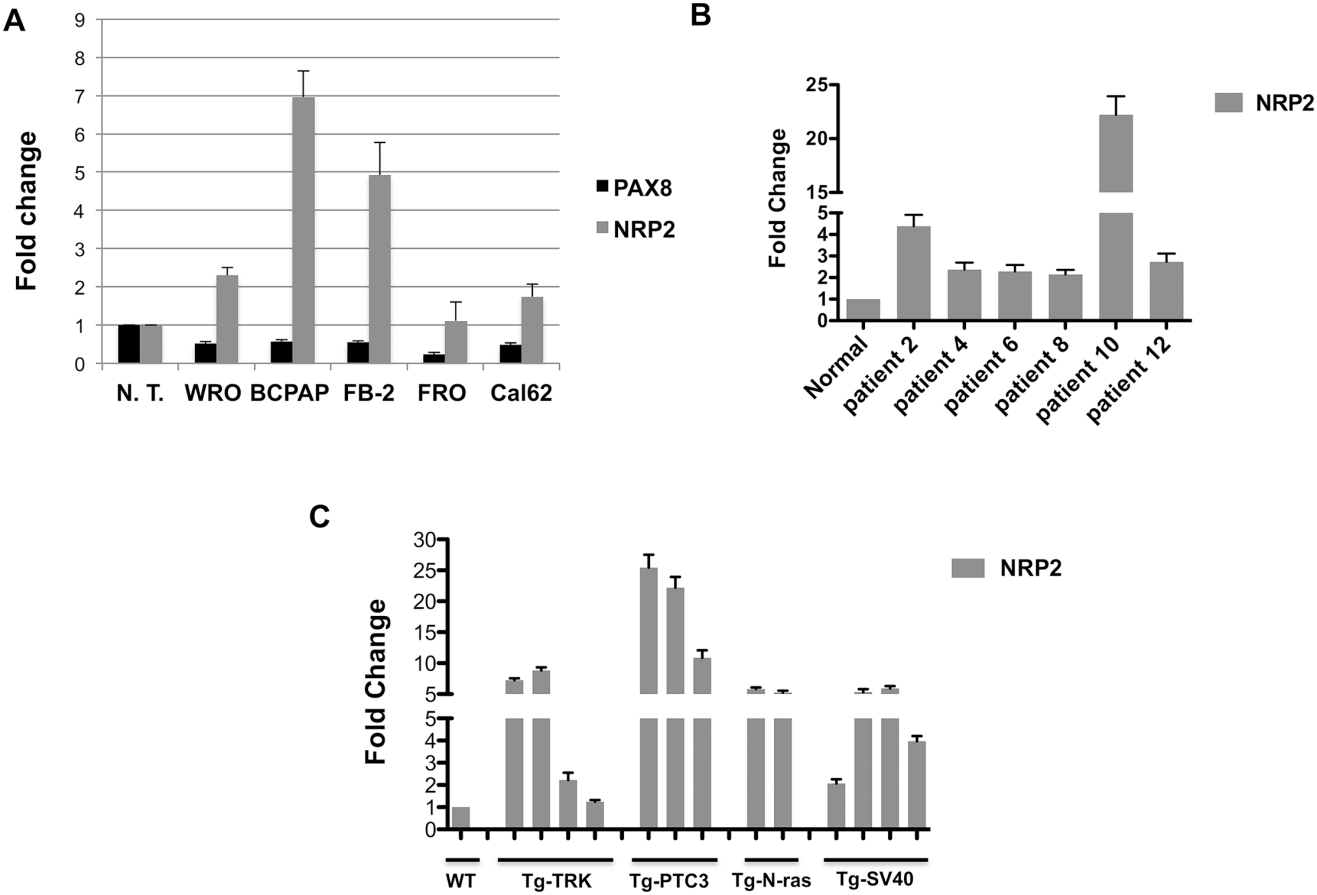
NRP2 expression in experimental models of thyroid carcinogenesis. (A) NRP2 and PAX8 expression was measured by qRT- PCR in five human thyroid cancer cell lines: WRO from follicular thyroid cancer, FB-2 and BCPAP from papillary thyroid carcinoma, Cal62 and FRO from anaplastic thyroid carcinoma. RNA from six non-pathological thyroids was used as control. ABL1 was used as reference gene; results are reported as 2^-ΔCt^. Statistical analysis uses t-test (p ≤ 0.05). (B) qRT-PCR analysis was performed on total RNA prepared from 6 tissue specimens of PTC. A pool of six normal thyroid tissues (N.T.) was used as control. Statistical analysis uses t-test (p ≤ 0.05). (C) qRT-PCR analysis was performed on total RNA prepared from thyroid carcinomas developed in Tg-TRK, Tg-PTC3, Tg-N-ras and Tg-SV40 transgenic mice expressing TRK, RET/PTC3, N-ras and SV40 T-antigen under the transcriptional control of the Tg promoter. Relative quantities of NRP2 mRNA were normalized to cyclophilinA as reference gene. Each amplification was performed in duplicate and the data are expressed as fold change with respect to the normal mouse thyroid tissues used as a control, whose value was set at 1.0. Statistical analysis uses t-test (p ≤ 0.05).

To evaluate the involvement of NRP2 in thyroid carcinogenesis and to strengthen the evidence for the relationship between PAX8 and NRP2 expression, we determined the mRNA levels of NRP2 in the WRO, BCPAP, FB-2, FRO and Cal62 cells derived from follicular, papillary and anaplastic thyroid carcinoma and in a pool of six normal thyroid tissues as control. As shown in Fig 2A, the reduction of PAX8 expression clearly corresponds to a significant increase of NRP2 expression. To further reinforce our observation, we evaluated NRP2 mRNA levels in 6 tissue specimens of PTC by quantitative RT-PCR. As control, we used a pool of six normal thyroid tissues. All the PTC samples analyzed express much higher NRP2 mRNA levels in comparison to normal thyroids indicating that a deregulation of NRP2 expression occurs in PTC (Fig 2B). A similar result was obtained when we evaluated NRP2 expression in thyroid neoplasia developed in transgenic mouse lines expressing different oncogenes under the transcriptional control of the thyroglobulin promoter. In particular, we analyzed transgenic mice carrying TRK and RET/PTC3 oncogenes, which develop papillary thyroid carcinomas (PTC) [57, 58] and N-ras mice that develop thyroid follicular tumors that undergo dedifferentiation, predominantly follicular thyroid carcinoma (FTC) [59]. Anaplastic thyroid carcinomas (ATC) were obtained from mice carrying the simian virus 40 large T antigen [60]. Fig 2C shows the results of the qRT-PCR analysis with expression values normalized for cyclophilin-A expression and reported as fold change with respect to the normal mouse thyroid used as a control. NRP2 expression is significantly increased in all the PTC and follicular thyroid carcinoma samples derived from TRK, RET/PTC3, and N-ras mice and in the anaplastic thyroid carcinoma samples derived from the simian virus 40 large T mice. These results clearly indicate that the absence of PAX8 affects NRP2 expression and further support a role for PAX8 in the transcriptional repression of this gene.

### PAX8-mediated NRP2 inhibition in FB-2 cells leads to reduced cell proliferation and suppresses cell migration and invasion

**Fig 3:**
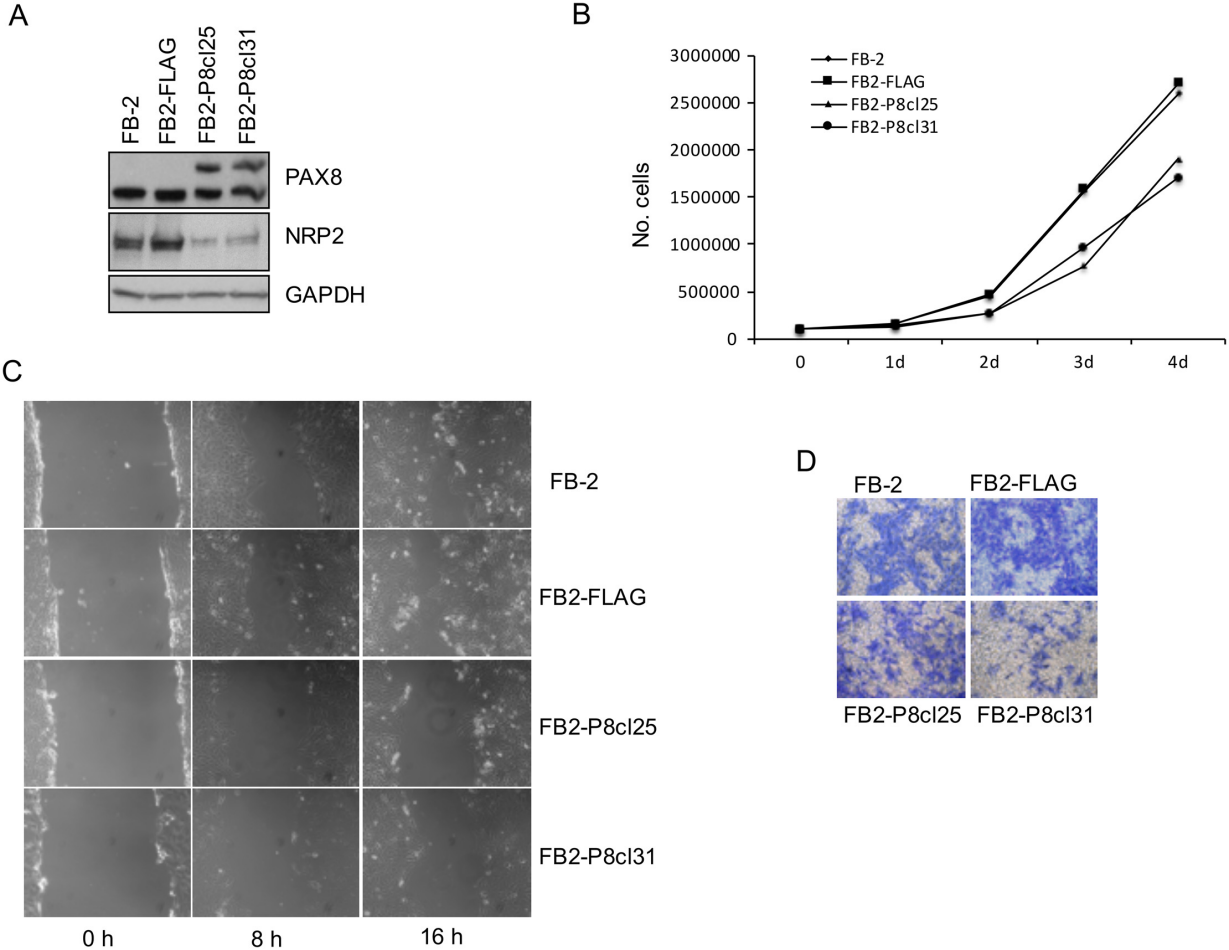
NRP2 downregulation in FB-2 cells inhibits cell proliferation, migration, and invasion. (A) NRP2 downregulation in PAX8 clones was analyzed by Western blot with a specific anti-NRP2 antibody. (B) Growth curves of FB-2, FB2-FLAG, FB2-P8cl25 and FB2-P8cl31 cells are shown. Triplicate of 8 x 10^4^ cells were seeded into 60-mm plate. Cell numbers were counted on days 1, 2, 3 and 4 after seeding. (C) Wound-healing migration assay for FB-2, FB2-FLAG, FB2-P8cl25 and FB2-P8cl31 cells were performed. The healing of the wounds by migrating cells was imaged at time 0, 8 and 16 h. (D) Matrigel invasion assay of FB-2, FB2-FLAG, FB2-P8cl25 and FB2-P8cl31 cells. The cells were seeded on 8 μm pore size Transwell filters and allowed to migrate toward 10% fetal bovine serum. After 16 h, the upper surface of the filter was wiped clean and cells on the lower surface were stained and photographed. This figure is representative of three independent experiments.

To examine whether high levels of NRP2 could directly contribute to the tumorigenicity of thyroid cancer cells, we analyzed whether NRP2 downregulation, mediated by PAX8 re-expression, was able to modify the oncogenic properties of FB-2 cells. To this aim, FB-2 cells were stably transfected with a PAX8 expression vector (FLAG-PAX8) or with the empty vector (pCEFL-FLAG) and the over-expression of PAX8 in the stable clones was validated by immunoblot analysis (data not shown). As expected, the upregulation of PAX8 in two independent representative clones (FB2-P8cl25 and FB2-P8cl31) strongly reduces NRP2 expression compared to parental cells or cells transfected with the control vector (Fig 3A).

To determine whether NRP2 reduction was able to modify the proliferation properties of FB-2 cells, we evaluated the growth rate of FB-2 clones FB2-P8cl25 and FB2-P8cl31. Cells were grown and counted each day for 4 days to establish cell proliferation curves. The analysis shows that the proliferation rate of these two clones is significantly lower than that of the parental or backbone vector-transfected FB-2 cells (Fig 3B). To further study the role of PAX8 in cell migration and invasion, wound healing and transwell assays were performed. In the wound healing assays, we compared the cell motility of the FB2-P8cl25 and FB2-P8cl31 stable clones with that of FB2-FLAG cells. After 8 hours, the area of the wound is significantly re-covered by migrating FB2-FLAG cells, and after 24 hours the wound area has almost been completely re-covered. In contrast, the motility of the two overexpressing PAX8 clones is significantly decreased; suggesting that NRP2 downregulation strongly reduces the migration ability of FB-2 thyroid cancer cells (Fig 3C). Lastly, we performed a migration assay in which cells were seeded in serum-free medium on the top chamber of a 2-chamber transwell cell culture plate. Colorimetric evaluation of the cells migrated to the lower chamber revealed fewer migrated FB2-P8cl25 and FB2-P8cl31 cells with respect to FB2-FLAG cells (Fig 3D).

All together, our results indicate that NRP2 is involved in cell migration and invasion capabilities of FB-2 thyroid cancer cells.

### NRP2 downregulation reverses the EMT phenotype of thyroid cancer cells

During tumor progression, cancer cells can acquire motility and invasive ability by initiating the EMT process, which can potentially lead to distant metastases. Interestingly, NRP2 was recently reported to play an essential function in promoting EMT [35]. Having previously established that NRP2 downregulation decreases proliferation, motility and invasion, we hypothesized that it could also revert the mesenchymal phenotype of the FB-2 cells.

To test this hypothesis, we analyzed by qRT-PCR the expression levels of epithelial and mesenchymal markers in the FB2-P8cl25 and FB2-P8cl31 clones.

**Fig 4:**
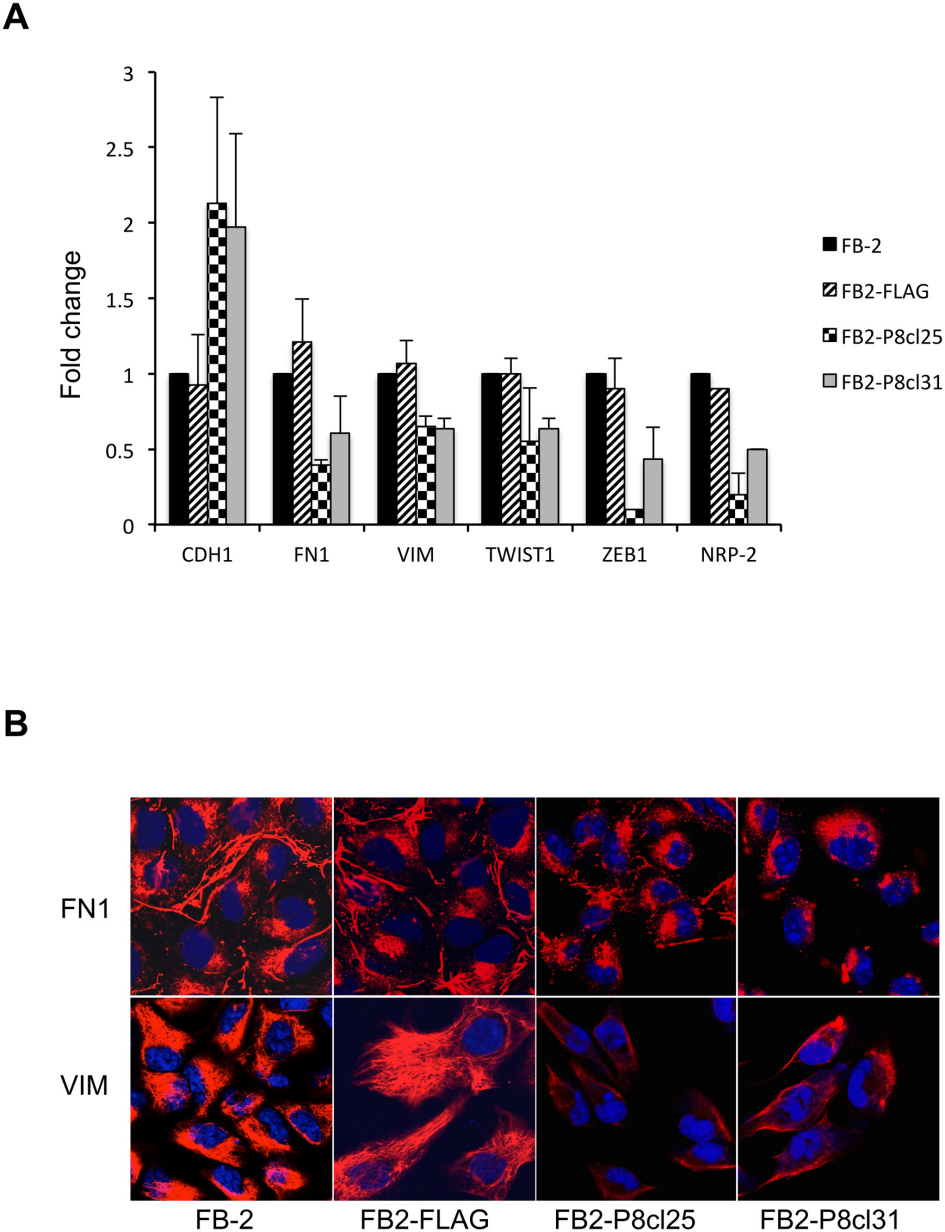
Effect of NRP2 downregulation on EMT in thyroid cancer cells. (A) qRT-PCR analysis was performed on total RNA prepared from wild-type FB-2 cells and individual Pax8 stable clones. The expression of E-cadherin, fibronectin-1, vimentin, twist-1 and zeb-1 was measured. The values are means ± SD of three independent experiments in duplicate, normalized by the expression of ABL1 and expressed as fold change with respect to the expression in FB-2 control cells, whose value was set at 1.0. Statistical analysis uses t-test (p ≤ 0.05). (B) Indirect immunofluorenscence showing vimentin and fibronectin-1 staining in control cells and Pax8 stable clones.

As shown in Fig 4A, the mRNA level of the epithelial marker Ecadherin (CDH-1) is upregulated, whilst the expression of the mesenchymal markers fibronectin-1 (Fn-1), vimentin (VIM), twist-1 and zeb-1 is downregulated with respect to the FB-2 parental cell line, suggesting that downregulation of NRP2 is also able to block EMT.

We also detected by immunofluorescence the presence of the mesenchymal markers vimentin and fibronectin-1 in wild-type FB2 cells and in the two clones. As shown in Fig 4B, the cytoskeletal protein vimentin is organized in a rich network of filaments surrounding the cell nucleus in wild-type FB2 cells while in the FB2-P8cl25 and FB2-P8cl31 cells vimentin staining is reduced together with a dramatic decrease of the filament structure. In addition, a rich deposition of the extracellular matrix protein fibronectin-1 is observed in control cells which is almost competely lacking in the two clones (Fig 4B).

## Discussion

Transcription factors play a pivotal role in the determination and maintenance of the cellular phenotype. The activity of transcription factors is in fact considered as the main switch to regulate gene expression. The differentiation program of thyroid follicular cells (TFCs), by far the most abundant cell population of the thyroid gland, relies on the interplay between sequence-specific transcription factors and transcriptional coregulators with the basal transcriptional machinery of the cell. A correct process of thyroid organogenesis, morphogenesis, and follicular cell differentiation is required for the maintenance of ordered architecture and function of the differentiated thyroid gland. However, the molecular mechanisms leading to the fully differentiated thyrocyte are still the object of intense study. Many data suggest that the development of the embryonic thyroid gland and its migration require an interplay between four transcription factors, Pax8 (TTF-1/Nkx2.1), Foxe1, Hhex, and Nkx2.5 [61, 62]. PAX8 is a member of the Pax genes family, a family of genes that plays an essential role in body patterning during development. Specifically, PAX8 has been demonstrated to be required both for the morphogenesis of the thyroid gland and for the maintenance of the thyroid-differentiated phenotype. Interestingly, in *Pax8* knockout mice the thyroid gland is barely visible and lacks the follicular cells [40–43]. In humans, patients carrying mutations in the PAX8 gene suffer from congenital hypothyroidism [46–49]. Although the relevance of the role of PAX8 is becoming more and more clear, its downstream targets are still poorly described. In this paper, we show that PAX8 negatively regulates the expression of Neuropilin-2 (NRP2) by directly binding to a region of the NRP2 promoter. It has been reported that a progressive decrease of PAX8 levels occurs in thyroid tumors from follicular adenoma to differentiated carcinoma and then to anaplastic carcinoma, which parallels the progressive dedifferentiation and increasing malignancy of thyroid tumors [63]. Here, we demonstrate that in mouse thyroid carcinomas resembling papillary, follicular, and anaplastic carcinomas, the expression of NRP2 is strongly increased. The same happens in the thyroid tissues from patients affected by papillary carcinomas and in the human thyroid cancer cell lines that we analyzed. The increased expression of NRP2 parallels the decreased expression of PAX8 allowing us to speculate that this transcriptional factor, mostly shown to be a transcriptional activator, is also able to act as a transcriptional repressor in agreement with other data already available in the literature[64, 65].

Many malignant tumor cell lines express NRP2 and this appears to contribute to their aggressiveness. Overexpression of NRP2 is shown to enhance growth, correlate with invasion and is associated with poor prognosis in various tumor types, especially those of epithelial origin [9, 10]. To investigate the role of NRP2 in thyroid cancer, we have chosen the FB-2 cell line and selected stable cell clones overexpressing PAX8. Our results indicated that PAX8-mediated NRP2 downregulation elicits a dramatic effect on FB-2 cell growth, inhibits the invasion rate of these cells through the Matrigel and reduces the migration rate in wound healing assays.

The epithelial-mesenchymal transition (EMT) is a biologic process that allows a polarized epithelial cell, which normally interacts with basement membrane via its basal surface, to undergo multiple biochemical changes that enable it to assume a mesenchymal cell phenotype, which includes enhanced migratory capacity, invasiveness, elevated resistance to apoptosis, and greatly increased production of ECM components. The completion of an EMT is signaled by the degradation of underlying basement membrane and the formation of a mesenchymal cell that can migrate away from the epithelial layer in which it originated. Interestingly, Grandclement et al. identified an important function for NRP2 in promoting EMT [35]. To further characterize NRP2 effects on cell migration and invasion we analyzed the expression level of some master regulators of EMT in FB-2 cells stably transfected with PAX8. Here, we report that the downregulation of NRP2 significantly induces CDH1 expression while inhibits all the mesenchymal markers that we tested, suggesting that this protein might represent a possible new target for preventing thyroid tumor invasion and metastasis. We believe that a detailed knowledge of NRP2 transcriptional regulation could be relevant to explore alternative approaches to target its expression in pathological conditions.

## Supporting Information

S1 Fig Quantitation of the wound-healing and invasion assays. (TIF)

S1 Table Sequences of the oligonucleotide primers used in the qRT-PCR analysis. (DOCX)
