# Supplementary figures and images for: Neuropilin-2 Is a Newly Identified Target of PAX8 in Thyroid Cells
Source: PLoS One. 2015 Jun 1;10(6):e0128315. doi: 10.1371/journal.pone.0128315 (PMC4451263; doi:10.1371/journal.pone.0128315)

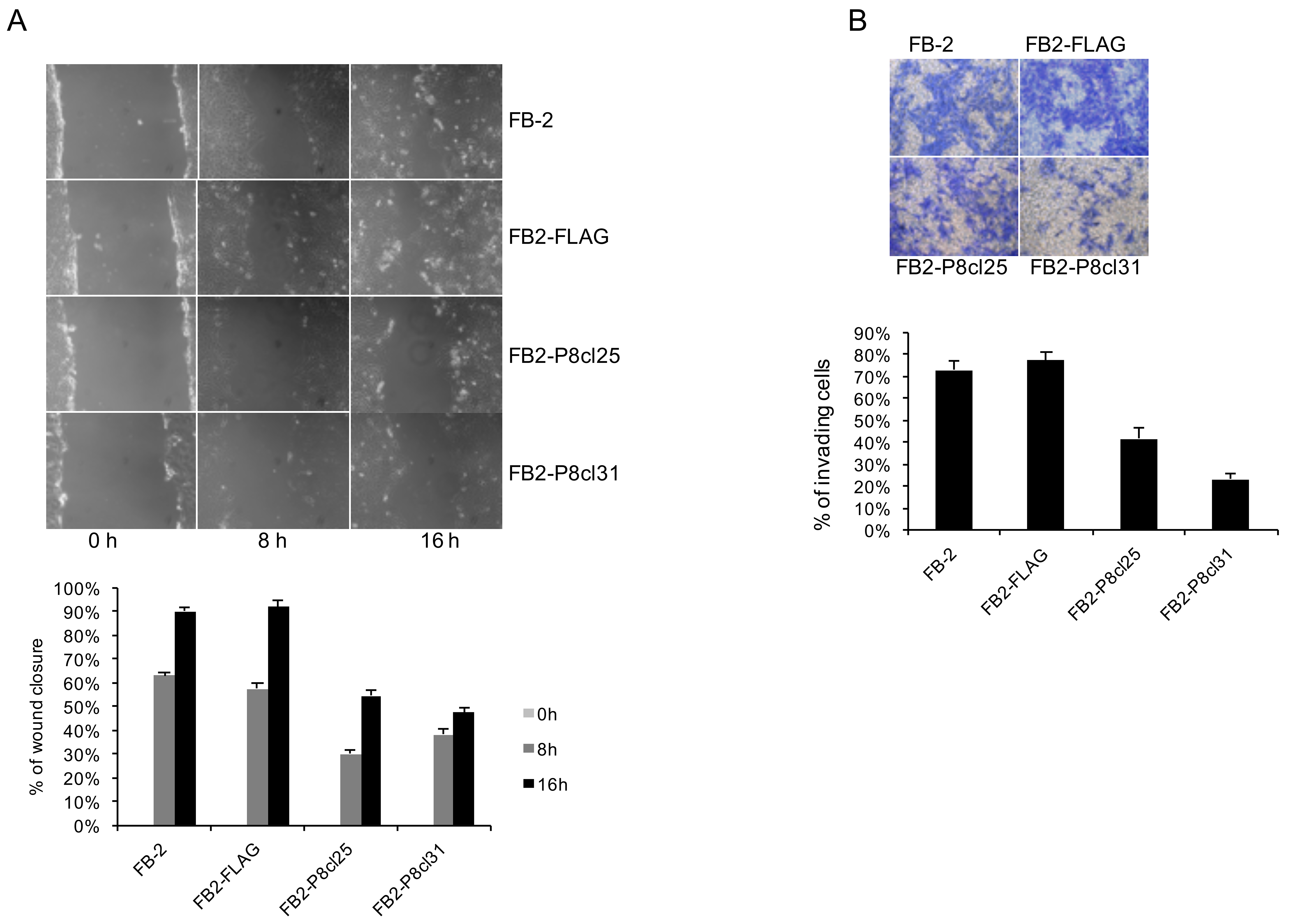

Supplement: S1 Fig — (TIF) [file pone.0128315.s001.tif]
