# Supplementary material for: Neuropilin-2 Is a Newly Identified Target of PAX8 in Thyroid Cells
Source: PLoS One. 2015 Jun 1;10(6):e0128315. doi: 10.1371/journal.pone.0128315 (PMC4451263; doi:10.1371/journal.pone.0128315)
Supplement: S1 Table — (DOCX) [file pone.0128315.s002.docx]

| **GENE NAME** | **FORWARD PRIME 5’-3’** | **REVERSE PRIMER** **5’-3’** |
| --- | --- | --- |
| h-NRP2 | ctggaaagagccaccttctcc | accgggagaggtgatatagcc |
| h-PAX8 | cccttccaacacgccact | ctgctttatggcgaagggtg |
| h-ABL | tggagataacactctaagcataactaaagg | gatgtagttgcttgggaccca |
| r-NRP2 | gagatcccaccaacctagcc | gcggatcttgctggcttctc |
| r-b-actin | ggcaatgagcggttccgatg | atggtggtgccaccagacag |
| r-PAX8 | cagctatgcctcttccgctatt | tgtggctgtaggcattgcc |
| h-FN1 | agcggacgcatcacttgcac | tgcactggagcaggtttcctc |
| h-VIM | cgggagaaattgcaggaggag | caaggtcaagacgtgccagag |
| h-CDH1 | cgagagctacacgttcacgg | ctttgaatcgggtgtcgaggg |
| h-TWIST1 | gtcttacgaggagctgcagac | cagcgtggggatgatcttcc |
| h-ZEB1 | ccagaagccacgatccagac | actgcatgaccatcgcgttcc |
| m-NRP2 | gatcccacaaacctagcccc | ggtggatcttgctggcttctc |
| m-Cyclophilin A | gcagacaaagttccaaagacag | caccctggcacatgaatcc |
